# Risk for Hereditary Neoplastic Syndromes in Women with Mismatch Repair-Proficient Endometrial Cancer

**DOI:** 10.3390/genes14111999

**Published:** 2023-10-26

**Authors:** Jennifer Thalita Targino dos Santos, Reginaldo Cruz Alves Rosa, Alison Luis Eburneo Pereira, Alan Vinicius Assunção-Luiz, Bruna Tavares Bacalá, Victor Evangelista de Faria Ferraz, Milena Flória

**Affiliations:** 1 Ribeirão Preto College of Nursing, University of São Paulo, Ribeirão Preto 14040-901, SP, Brazil; thalitatargino@usp.br (J.T.T.d.S.); assuncao@alumni.usp.br (A.V.A.-L.); brunabacala@hotmail.com (B.T.B.); 2 Department of Genetics, Ribeirão Preto Medical School, University of São Paulo, Ribeirão Preto 14040-901, SP, Brazil; reginalldo.usp@gmail.com (R.C.A.R.); alisongonpereira@alumni.usp.br (A.L.E.P.); vferraz@usp.br (V.E.d.F.F.)

**Keywords:** uterine neoplasms, lineage, patterns of inheritance, risk, family history

## Abstract

Endometrial cancer (EC) is a prevalent malignancy in women, and those who are proficient in the DNA mismatch repair (pMMR) pathway may have a family history (FH) that meets the criteria for a hereditary neoplastic condition (HNS). This study aimed to estimate the risk of HNS in women with pMMR endometrial tumors by analyzing their FH. To achieve this, we collaborated with a primary study and collected FH information by telephone. The final sample comprised 42 women who responded to the Primary Screening Questionnaire. Their family pedigrees were drawn and categorized according to internationally standardized criteria for the risk of HNS. Results showed that 26 women (61%) were found to be at risk for HNS, with Bethesda criteria being met by 23%, Amsterdam criteria by 15%, and 4% met the attenuated familial adenomatous polyposis criteria. Our results emphasize the importance of FH and the need to encourage healthcare professionals to collect and document FH more frequently, even if it is self-reported. By identifying individuals with HNS, we can improve their outcomes and reduce the burden of cancer in families with a predisposition to cancer.

## 1. Introduction

Endometrial cancer (EC) is the most common gynecological malignancy associated with Lynch syndrome (LS) [1]. Hereditary factors account for 2–6% of all EC cases and result from germline mutations that affect the function of the four key genes in the mismatch repair (MMR) pathway, *MLH1*, *MSH2*, *MSH6*, *PMS2*, or deletions at the final exons of *EPCAM* [1,2]. Women with LS have a 28–60% chance of developing EC, depending on the affected gene [3]. Although colorectal and EC are the most common tumors associated with LS, it has also been linked to tumors in other organs [2]. Moreover, other diseases such as breast cancer have been associated with LS, but more research is needed to explore these associations [4]. Families with the same tumor spectrum as LS but no MMR germline mutations are categorized as “familial colorectal cancer type X (FCCTX),” which meets the Amsterdam criteria [2]. An advanced comprehension of the clinical and histological manifestations associated with hereditary non-polyposis colorectal cancer (HNPCC) allowed the formulation of the Bethesda Guidelines. These guidelines systematically outline specific criteria for the identification of colorectal cancer, wherein criteria were delineated for identifying colorectal tumors eligible for microsatellite instability (MSI) testing [2]. Germline mutations in other genes, such as *TP53* or *PTEN*, cause hereditary EC and are associated with Li-Fraumeni and Cowden hereditary neoplastic syndromes [5,6]. Having a first-degree relative with EC increases a woman’s risk of developing the disease by two-fold [1,7].

It is worth noting that 20% of all cancer patients may meet the criteria for HNS based on their family history [8,9,10]. However, recording a family history is not typically prioritized [11]. EC is often initially considered sporadic because it is diagnosed after the age of 50 and does not exhibit morphological or molecular abnormalities in the DNA repair system [12,13,14,15]. Nonetheless, EC may have hereditary components that require investigation, even in patients with proficient mismatch repair (pMMR) tumors [12]. Therefore, identifying and assessing the personal and family history of cancer in women diagnosed with EC, initially classified as sporadic, is critical. The aim of this study is to assess the risk for hereditary neoplastic syndromes in women with EC who are proficient in MMR by analyzing their family history. 

## 2. Materials and Methods

This is a cross-sectional study utilizing a sample obtained from a primary study that evaluated EC patients in terms of proficiency of the MMR in the Brazilian population. The primary study retrospectively analyzed 127 EC patients who underwent surgery at a university general hospital in Southeast Brazil between 2005 and 2017. Among these patients, 58 women were identified as pMMR, using immunohistochemistry, indicating a negative family history of cancer and no risk of hereditary neoplastic syndromes (HNS), thus comprising our initial sample [16]. 

The Scientific and Research Committee of the Ribeirão Preto Medical School of the University of Sao Paulo, Brazil (HC-FMRP-USP) approved the research protocol and consent form for this study (protocol number 1.578.206/2016). Participants in the primary study provided written consent to collect information on their family history of cancer, which is used in the present study [16].

The inclusion criteria were as follows: in addition to having pMMR endometrial tumors, participants had to be over 18 years old and have a working phone contact, which was the method used to collect data. Those with obsolete or non-existent phone numbers recorded in the hospital database, as well as those who could not be contacted after 15 attempts, were excluded from the study.

From April to July 2018, the primary researcher attempted to contact and collect the personal cancer history (PH) and family history (FH) of the 58 participants. FH of cancer in family members of at least three generations was evaluated. PH included current age and age at diagnosis, the occurrence of other primary cancers, and recurrence. Clinical data, such as histological subtype, tumor grade, and staging according to the International Federation of Gynecology and Obstetrics (FIGO), and numerical variables, such as age at cancer diagnosis, were obtained from the primary study’s database and hospital records, including electronic medical records and phone contacts of participants [16].

A single, fully qualified interviewer, the main researcher, conducted all the interviews. She used the telephone approach, contacted patients diagnosed with EC, and explained the project’s goals. After reading the consent form and obtaining verbalized consent, the ‘Primary Screening Questionnaire’ (PSQ—Supplementary Material S1) was administered. It comprises a previously validated instrument with three ‘Yes’ or ‘No’ questions and one open-ended question [10]. The first question pertained to the personal history of cancer before the age of 50. The second question included sub-items about the presence of breast, bowel, and/or ovarian cancer in first- or second-degree relatives before the age of 50. The third question, open-ended, inquired about the presence of cancer in three or more first- or second-degree relatives before the age of 50.

To collect information on cancer cases that were not initially addressed in the PSQ, direct questioning of each family member was performed. This allowed for data collection from at least three generations, involving affected and healthy people, in both the maternal and paternal lineages [17,18] Using the PedigreeDraw software (Genial Genetic Solutions, discontinued on 1 May 2020) in its free edition, the information gathered during the interviews was exclusively from the proband. This data was then utilized to construct the pedigrees. These pedigrees were separately assessed by the main researcher and, subsequently, by two partner oncogeneticists. These professionals collaborated to develop plausible diagnostic hypotheses regarding HNS after collecting and documenting the self-reported family history. Families were investigated to determine whether they met the Amsterdam criteria and the revised Bethesda criteria [19]. They were then categorized according to the National Comprehensive Cancer Network (NCCN) criteria for the risk of HNS.

The data were analyzed using descriptive statistics, and Fisher’s exact test, running on R, was employed to check for a statistical association between FIGO tumor stage, suspected HNS, and age at diagnosis, with *p*-values of less than 0.05 being considered significant.

## 3. Results

**Figure 1:**
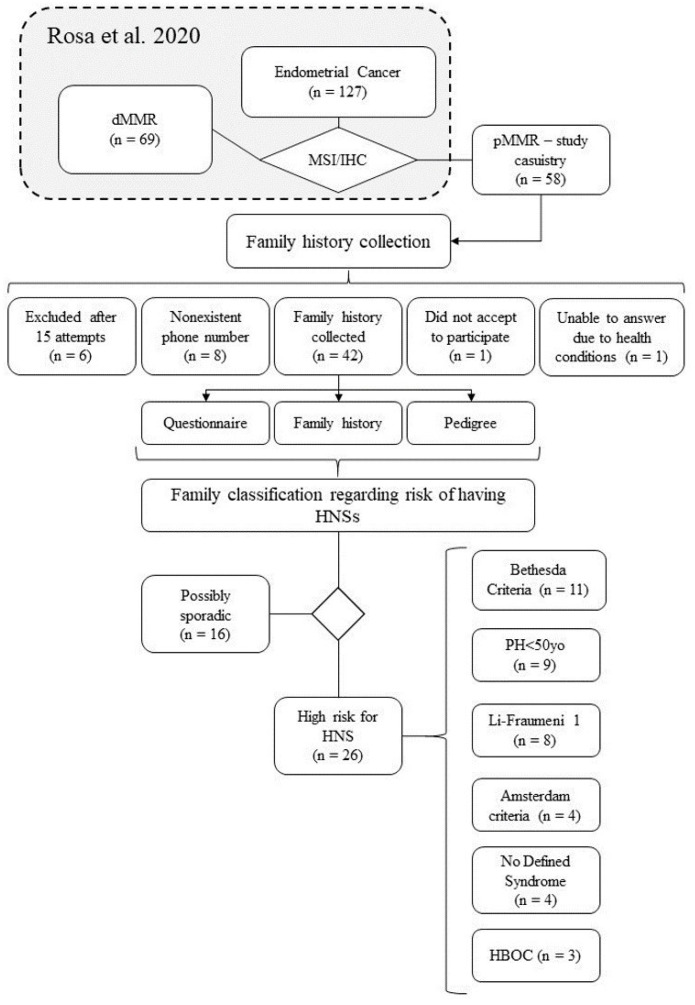
Workflow depicting the primary study’s casuistry (n = 127), the current study’s (n = 58), and the composition of our final sample (n = 42). The diagram also showcases the distribution of samples meeting the criteria for each listed HNS, with the acknowledgment that a single sample may fall under multiple syndrome criteria [16].

Initially, 58 women were deemed eligible for this study, out of which 43 patients were successfully contacted by phone, but one of them declined to participate. Ultimately, a final sample of 42 women was obtained. The study design flowchart is depicted in Figure 1. The average age at the time of EC diagnosis was 58.4 years, with a range of 33 to 91 years. Other pertinent features are shown in Table 1.

Of the 42 women who participated in the study, personal and family cancer history was collected. Out of these, 26 (61.9%) individuals met the primary questionnaire and National Comprehensive Cancer Network (NCCN) criteria (Supplementary Material), which put them at risk for a HNS, as shown in Table 2. Three families (7.14%) allowed access to data from up to five generations through the use of pedigree, while 15 families (35.71%) allowed access to data from up to the fourth generation, and 23 families (54.76%) allowed access to data from up to the third generation. Only one family (2.38%) recorded data for two generations.

In the studied population, twenty probands (47.6%) had at least one first-degree relative with cancer, 28 (66.7%) had second-degree relatives with cancer, and 16 (38.1%) had third-degree relatives with cancer. Seven probands (16.7%) had at least one afflicted first-degree relative with cancer under the age of 50, six (14.3%) had second-degree relatives with cancer under the age of 50, and seven (16.7%) had third-degree relatives with cancer under the age of 50.

On average, three tumors were found in each family (SD = 2.01; minimum 0, maximum 8). Five cases (4.54% of all family members with neoplasms in the present study) of colorectal cancer (CRC), nine (8.18%) of breast cancer, and one case (0.91%) of ovarian cancer were observed to occur before the age of 50. Breast cancer (15.60%), prostate cancer (14.68%), and CRC (13.76%) were the most frequent types of cancer in the studied population, as presented in Table 3.

Pedigrees were examined to investigate whether any clinical criteria were present in the families. The average age of probands who met possibly sporadic cancer criteria was 62.7 years, whereas those suspected of having any HNS was 55.5 years. 

This study found that the average age of individuals in pedigrees meeting the Bethesda criteria was 58.7 years (n = 6), while those who met the Amsterdam criteria had an average age of 63 years. Women who met the criteria for hereditary breast and ovarian cancer (HBOC) syndrome had a mean age of 48 years (n = 3), while those who met the criteria for Li-Fraumeni type 1 had a mean age of 61.3 years (n = 6). Only one patient with attenuated familial adenomatous polyposis (aFAP) was identified, and this patient was 43 years old.

Of the total patients, the majority (n = 28; 58.3%) had IA FIGO staging EC. The study further observed that women with an HNS had a higher degree of FIGO staging than sporadic cases (Fisher’s exact test, *p* < 0.05). However, there was no significant association found between FIGO staging and women diagnosed before the age of 50.

## 4. Discussion

In addition to personal cancer history, family history information is one of the most effective tools for identifying individuals at an increased risk of developing cancer [20,21,22]. This analysis enables health professionals to refer people to genetic counseling programs, perform more intensive screenings, follow up at a younger age, and offer genetic testing for HNS susceptibility genes [23,24,25].

Results of this study revealed that despite having pMMR tumors, 61.9% of the women investigated had at least one criterion for HNS based on FH and PH. The classification of such tumors would reduce the possibility of association with Lynch syndrome.

According to our data, 15% of the women in our study met the Amsterdam II criteria, which is indicative of familial colorectal cancer type X (FCCTX) [26,27]. FCCTX is a clinically heterogeneous group that encompasses several hereditary disorders and cancer aggregations [28,29,30]. Previous research has identified clinical and pathological differences between FCCTX and LS [31,32]. For example, FCCTX is associated with an older mean age at diagnosis of 50 to 65 years, compared to 40 years for LS [31,32]. This age difference was also observed in our study, where women with FCCTX had an average age of 63 years.

FCCTX includes several hereditary disorders and cancer aggregations with various candidate target genes, including *BRCA2* [33], *SEMA4A* [30], *KRAS* [27], *BRAF* [34], *APC* [33], and *BRIP1* [35]. In one of the pedigrees studied, several members who met the FCCTX criteria had a history of intestinal polyps. The *BMPR1A* gene has been previously associated with FCCTX [33], as well as with juvenile polyposis in 20% of cases and hereditary mixed polyposis syndrome in 50% of cases [36,37]. However, BRCA2 seems to have a more significant role in FCCTX cases [38]. Garre [39] reported the first evidence of a link between germline BRCA2 mutations and FCCTX in individuals who met the criteria. For instance, one of the probands in this study who met the FCCTX criteria had two first-degree relatives with prostate cancer, one second-degree cousin with prostate cancer, one third-degree relative with colorectal cancer, and four third-degree relatives with colorectal cancer. Although the existing data do not provide direct evidence of a link between *BRCA1* and *BRCA2* gene mutations and diagnoses of colorectal and EC, these findings support the notion that these mutations predispose to a broader spectrum of cancers than previously believed [38]. 

**Figure 2:**
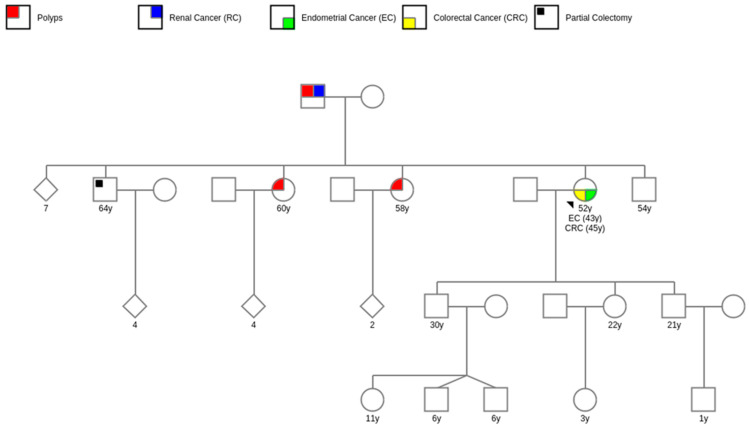
Pedigree example of a proband with two primary cancers.

Two of the probands exhibited metachronous tumors, one had metachronic colorectal cancer and the other had thyroid cancer (Figure 2). Second primary malignancies may be linked to extensive medical surveillance after the initial diagnosis; treatments produced by exposure to X-rays and antineoplastic medicines; environmental factors, and high-risk genetic variants associated with HNS [40].

One proband met the Bethesda criteria for Lynch syndrome (LS), and six others had family members who also met the criteria. While the Bethesda criteria are more sensitive than the Amsterdam criteria, they are often used for LS screening [41,42]. However, up to 50% of individuals with LS do not meet the revised Bethesda criteria [19]. Moreover, since the research on the Bethesda criteria was primarily focused on colorectal cancer, their applicability to EC remains uncertain [43].

Regarding other families that met the criteria for any HNS, 22% (n = 8) of these were identified as families that met the criteria for Li Fraumeni Syndrome. All families that met the criteria for Li-Fraumeni corresponded to Li-Fraumeni-Like Type 1, as proposed by Eeles [44]. The average age of probands who met the Li-Fraumeni criteria was 61.3 years, which is considered late when compared to previous studies that estimate a cumulative risk of up to 90% for the development of a wide range of cancers before 45 years in this syndrome [45]. Our findings, however, are explained by Achatz [46], who discovered a particular mutation in the *TP53* gene (R337H) in southern and southeastern Brazil, which was linked to a founder effect. This mutation has its unique features, such as lower penetrance when compared to conventional Li-Fraumeni syndrome; as a result, the diagnosis is delayed until later in life. The investigation of the variation in this study’s probands may help to explain this finding.

One noteworthy characteristic of our study population was the presence of 33% of participants diagnosed with EC before the age of 50. Typically, EC affects the majority of women over 50, with a mean age of about 60, and is rare in women under 40 [47,48]. Most studies have reported that younger EC patients tend to have lower-grade histological subtypes. In line with these previous findings, our investigation revealed a correlation between lower-grade cancer and staging in women diagnosed with EC before age 50 [48,49,50,51].

Individuals with suspected HNS were found to have a higher tumor grade compared to those with sporadic cases. These findings are consistent with previous studies, such as Carcangiu’s investigation in 2010, which reported that the proportion of grade 3 tumors was 46.1% among patients with HNS, compared to 11.3% among controls. Similar results have been observed in other syndromes, including Cowden syndrome, hereditary breast and ovarian cancer (HBOC), and Li-Fraumeni syndrome [52,53].

While FH of cancer may not meet the specific criteria for HNS in some cases, it may still warrant adjustments in cancer screening. In our study, 15% of families exhibited a cancer family cluster, which necessitates more vigilant monitoring than sporadic cancer cases, even if the individuals do not qualify for genetic testing for known susceptibility genes [54]. Moreover, family clusters often reflect non-genetic factors that are shared among family members, such as dietary habits, social and environmental exposures, as well as cultural beliefs and attitudes, that span multiple generations [24]. Hence, promoting health education and lifestyle interventions are crucial cancer prevention strategies for these families [55].

Our study found that several probands had a family history of colorectal and EC, with four having a first-degree relative with colorectal cancer, two having a first-degree relative with EC, and three having a second-degree relative with EC. Similar observations were reported by Cook [13], while Bharati [14] and Win [56] found that women with a first-degree relative diagnosed with colon or EC had a higher risk of developing EC themselves. These results suggest that EC may have a genetic component that runs in families, although environmental factors shared by family members may also play a role, either alone or in combination with genetic factors (gene-environment interaction) [56].

One potential limitation of our study is that it was conducted entirely over the phone. However, studies such as Campacci [10] have validated the use of the PSQ instrument to identify families at risk for HNS via telephone contact, finding no significant differences in applicability compared to in-person use. Similarly, Joseph [57] utilized a telephone survey to identify individuals who may benefit from genetic counseling. In fact, he found that telephone counseling can be used effectively to reduce geographic barriers and increase access to individuals without causing long-term negative psychosocial consequences. Therefore, while in-person counseling may be preferred in some cases, telephone counseling can be a useful alternative that enables increased access to care.

Our study provides important insights into the significance of collecting family history information in pMMR EC cases. The findings from our investigation, as well as other similar studies, highlight the critical role of FH in identifying individuals and families at increased risk of developing EC [12,13,14]. Our results also suggest that MMR gene mutations may account for only a small fraction of the overall familial risk of EC [13]. By identifying these high-risk patients and their families, our study highlights the importance of personalized cancer screening and prevention strategies, as well as tailored treatment options, including targeted therapies and prophylactic surgeries [3,4]. Overall, our study emphasizes the need for a comprehensive approach to the management of EC that incorporates family history data and individual risk assessments.

## 5. Conclusions

The results of this study demonstrate that, despite the advancements in genomic technologies and an improved understanding of genetic testing, FH remains a critical source of risk information for HNS, which extends beyond genetic susceptibility. Therefore, it is advisable that individuals and their families be evaluated based on their personal and familial history to identify potential HNS risks.

Identifying individuals with possible HNS enables healthcare professionals to implement preventive measures and intervene not only for an individual but also for the entire family. In addition, FH has been demonstrated to be a cost-effective and efficient technique for identifying individuals at an increased risk of developing cancer, particularly in Brazil where the majority of the population lacks access to health insurance, and genetic testing is financially unfeasible.

In order to enhance the prevention and treatment of EC in women pMMR, we suggest conducting additional studies that employ molecular and genetic approaches to investigate HNS. These advancements may lead to the identification of cost-effective techniques for identifying women at risk and improving the prevention and treatment of this neoplasm.

## Figures and Tables

**Table 1:** Sample characterization according to age, tumor histology, FIGO staging and myometrial invasion.

	Total N = 42 (100%)	High Risk N = 26 (100%)	Low Risk N = 16 (100%)
<50	6 (14.3%)	6 (23.1%)	-
50–59	16 (38.1%)	9 (34.6%)	7 (43.8%)
≥60	20 (47.6%)	11 (42.3%)	9 (56.3%)
Histology			
Endometrioid	39 (92.8%)	23 (88.5%)	16 (100%)
Papillary serous	1 (2.4%)	1 (3.8%)	-
Mixed	2 (4.8%)	2 (7.7%)	-
FIGO stage			
I	32 (76.2%)	19 (73.1%)	13 (81.2%)
II	5 (11.9%)	2 (7.7%)	3 (18.8%)
III	4 (9.5%)	4 (15.4%)	-
IV	1 (2.4%)	1 (3.8%)	-
Myometrial invasion			
Limited to endometrium	8 (17.1%)	6 (24.0%)	2 (12.5%)
Less than 50% of the myometrium	20 (48.8%)	11 (44.0%)	8 (50.0%)
More than 50% of the myometrium	13 (31.7%)	8 (32.0%)	6 (37.5%)

The percentages pertain to the total, high-risk, and low-risk groups, respectively, within the column. Source: Generated by the authors.

**Table 2:** Sample characterization according to NCCN criteria. Only samples with high risk are shown.

ID	01	02	03	04	05	06	07	08	09	10	11	12	13	14	15	16	17	18	19	20	21	22	23	24	25	26
AaD	33	34	39	43	44	45	48	48	49	51	55	56	58	59	59	59	60	62	64	64	65	65	66	68	71	91
FC																										
PC < 50																										
aFAP																										
Bethesda																										
AMST-II																										
HBOC																										
Li-Fraumeni																										
FIGO	IV	I	I	I	I	I	III	I	I	III	III	III	III	III	I	I	-	I	III	-	-	I	I	I	I	III
Other Cancer(CA, age)				Uterus, 45												Thyroid, 55					Breast, 60					
FBC < 50																										
FCC < 50																										
FOC < 50																										
+3C < 50																										

ID = sample ID. AaD = Age at diagnosis. FC = Familial cluster. PC < 50 = Personal history of cancer before 50 years-old. aFAP = attenuated familial adenomatous polyposis. AMST-II = Amsterdam-II criteria. FIGO = FIGO stage. FBC < 50 = Familial breast cancer before 50 years-old. FCC < 50 = Familial colorectal cancer before 50 years-old. FOC < 50 = Familial ovary cancer before 50 years-old. +3C < 50 = More than 3 relatives with any type of cancer before 50 years-old. Source: Generated by the authors.

**Table 3:** Distribution of malignant neoplasms in studied families based on their cancer history.

Type of Cancer	N (%)	Mean Age (St.Dev)	Type of Cancer	N (%)	Mean Age (St.Dev)
Breast	17 (15.6)	42.69 (14.10)	Leukemia	3 (2.75)	42.33 (33.23)
Prostate	16 (14.68)	70.13 (9.67)	Pancreas	3 (2.75)	71.33 (11.50)
Colorectal	15 (13.76)	57.47 (10.18)	Esophagus	2 (1.83)	71.00 (1.41)
Endometrium	7 (6.42)	55.14 (17.94)	Lymphoma	2 (1.83)	27.50 (6.36)
Head And Neck	6 (5.50)	31.75 (19.60)	Bone	1 (0.92)	40.00 (N/A)
Stomach	6 (5.50)	58.60 (6.11)	Kidney	1 (0.92)	-
Lung	5 (4.59)	58.00 (12.10)	Liver	1 (0.92)	43.00 (N/A)
Throat	5 (4.59)	56.25 (11.09)	Malanoma	1 (0.92)	40.00 (N/A)
Skin	4 (3.67)	60.00 (18.26)	Vulva	1 (0.92)	70.00 (N/A)

Source: Generated by the authors.

## Data Availability

The data used in this study contain sensitive information, including identifiable sample information. As such, access to the data will only be provided through formal requests and with appropriate ethical approvals in place. Requests for access should be directed to thalitatargino@usp.br, and will be evaluated on a case-by-case basis to ensure that all necessary ethical and legal requirements are met.

## Supplementary Materials

The following supporting information can be downloaded at: https://www.mdpi.com/article/10.3390/genes14111999/s1, Supplementary Material S1. Primary Screening Questionnaire (Translated from Brazilian Portuguese). Supplementary Material S2. Table S1—Criteria used to categorize the samples in the present study.
