# Supplementary material for: Risk for Hereditary Neoplastic Syndromes in Women with Mismatch Repair-Proficient Endometrial Cancer
Source: Genes (Basel). 2023 Oct 26;14(11):1999. doi: 10.3390/genes14111999 (PMC10671603; doi:10.3390/genes14111999)
Supplement: Supplementary file 1 [file genes-14-01999-s001.zip › genes-2670378-supplementary S1.pdf]

Supplementary Material S1. Primary Screening Questionnaire (Translated from Brazilian Portuguese)

Primary Screening Questionnaire

Patient name: ____

Mother's name: ____

Date of Birth: ____

ID number: ____

City and state: ____

Date: ____

Q1) Did you have cancer before age 50?

( ) Yes. What age? _______________________________________

( ) No

Q2) Are there among your close family members (parents, siblings, children, grandparents and uncles/aunts) any case of:

**Breast cancer before age 50:**	**( ) Yes**	**( ) No**
**Bowel cancer before age 50:**	**( ) Yes**	**( ) No**
**Ovarian cancer before age 50:**	**( ) Yes**	**( ) No**

Q3) Are there, among your close relatives (parents, siblings, children, grandparents and uncles/aunts), 3 or more cases of cancer before the age of 50?

____
