# Supplementary material for: Risk for Hereditary Neoplastic Syndromes in Women with Mismatch Repair-Proficient Endometrial Cancer
Source: Genes (Basel). 2023 Oct 26;14(11):1999. doi: 10.3390/genes14111999 (PMC10671603; doi:10.3390/genes14111999)
Supplement: Supplementary file 1 [file genes-14-01999-s001.zip › genes-2670378-supplementary S2.pdf]

Supplementary Table S1 – Criteria used to categorize the samples in the present study.

<table>
<tr><th colspan="2">Amsterdam II Criteria</th></tr>
<tr><td colspan="2">There should be at least 3 relatives with CRC; all the following criteria should be present:
- One should be a first-degree relative of the other 2
- At least 2 successive generations should be affected
- At least 1 CRC should be diagnosed before age 50
- Familial adenomatous polyposis should be excluded
- Tumors should be verified by pathological examination</td></tr>
<tr><th colspan="2">Revised Bethesda Criteria</th></tr>
<tr><td colspan="2">Tumors from individuals should be tested for MSI in the following situations:
1. Colorectal cancer diagnosed in a patient who is less than 50 years of age.
2. Presence of synchronous, metachronous colorectal, or other hereditary nonpolyposis colorectal cancer (HNPCC)-associated tumors, regardless of age.
3. Colorectal cancer with the MSI-H histology diagnosed in a patient who is less than 60 years of age.
4. Colorectal cancer diagnosed in one or more first-degree relatives with an HNPCC-related tumor, with one of the cancers being diagnosed under age 50 years.
5. Colorectal cancer diagnosed in two or more first- or second-degree relatives with HNPCC-related tumors, regardless of age</td></tr>
<tr><th colspan="2">Li-Fraumeni</th></tr>
<tr><td>Classic Li-Fraumeni:
Most common cancers
• Bone and soft tissue sarcoma
• Breast cancer
• Brain tumor
• Leukemia
• Adrenocortical carcinoma

- Multiple primary cancers common 50% cancers diagnosed before 30 years
- 28% breast cancers diagnosed before age 30 years and 89% before age 50 years
- No excess cancers above population rates beyond age 59 years</td><td>Li-Fraumeni-like:
Most common cancers
• Bone and soft tissue sarcoma
• Breast cancer
• Brain tumor
• Leukemia
• Adrenocortical carcinoma

- Prone to develop multiple primary cancers 63% cancers diagnosed before 30 years
- 32% breast cancers diagnosed before age 30 years and 97% before age 50 years
- Only 4 of 158 cancers in known or assumed carriers of germline p53 mutations occurred after age 59 years</td></tr>
<tr><th colspan="2">aFAP</th></tr>
<tr><td colspan="2">- Patients are clinically defined as having less than 100 synchronous colorectal adenomas.
- Present at a later age than patients with classical FAP and develop CRC 10 to 20 years older
- May be diagnosed as a single case in a family or, less frequently, it may be present in other family members, and it shows distinct pattern of inheritance.</td></tr>
<tr><th colspan="2">HBOC</th></tr>
<tr><td colspan="2">- Breast cancer at age 45 or younger in women
- Breast cancer at age 46–50 or younger in women and at least one close blood relative with breast cancer at any age or limited family history
- Triple negative breast cancer at age 60 or younger in women
- Breast cancer at any age in men</td></tr>
</table>

- Ovarian, fallopian tube, or primary peritoneal cancer
- Cancer in both breasts
- Pancreatic cancer or prostate cancer with Gleason score ≥7
- Breast, ovarian, pancreatic, or prostate cancer among multiple blood relatives
- Ashkenazi (Eastern European) Jewish ancestry
- A known BRCA mutation in the family
